# Public Health Economic Burden Associated with Two Single Measles Case Investigations — Colorado, 2016–2017

**DOI:** 10.15585/mmwr.mm6646a3

**Published:** 2017-11-24

**Authors:** Grace E. Marx, Jennifer Chase, Joseph Jasperse, Kaylan Stinson, Carol E. McDonald, Janine K. Runfola, Jillian Jaskunas, Donna Hite, Meghan Barnes, Michele Askenazi, Bernadette Albanese

**Affiliations:** ^1^Epidemic Intelligence Service, CDC; Division of Scientific Education and Professional Development, CDC; ^2^Tri-County Health Department, Greenwood Village, Colorado; ^3^Denver Public Health, Denver, Colorado; ^4^Colorado Department of Public Health and Environment.

During July 2016–January 2017, two unrelated measles cases were identified in the Denver, Colorado area after patients traveled to countries with endemic measles transmission. Each case resulted in multiple exposures at health care facilities and public venues, and activated an immediate and complex response by local and state public health agencies, with activities led by the Tri-County Health Department (TCHD), which serves Adams, Arapahoe, and Douglas counties. To track the economic burden associated with investigating and responding to single measles cases, personnel hours and supply costs incurred during each investigation were tracked prospectively. No secondary cases of measles were identified in either investigation. Postexposure prophylaxis (PEP) was administered to 31 contacts involving the first case; no contacts of the second case were eligible for PEP because of a delay in diagnosing measles disease. Public health costs of disease investigation in the first and second case were estimated at $49,769 and $18,423, respectively. Single measles cases prompted coordinated public health action and were costly and resource-intensive for local public health agencies.

## Patient A

On July 9, 2016, a male resident of Arapahoe County aged 14 months experienced a fever, with cough, coryza, and conjunctivitis reported during the subsequent 3 days. On July 12, the child developed a diffuse macular rash on the head that spread to the torso and legs. The child was evaluated by a pediatrician, who suspected hand-foot-mouth (Coxsackievirus) disease because of the presence of an ulcer in the oropharynx. The child had visited the pediatrician for pretravel counseling at age 10 months, before visiting India during March 30–June 30; however, measles-mumps-rubella (MMR) vaccine had not been administered.* The child was seen by a pediatrician again on July 13 and 14 with persistent fever, respiratory symptoms, and rash, along with lethargy and anorexia. The pediatrician referred the child to a local hospital emergency department on July 14. Upon evaluation, the child was transferred and admitted to a pediatric hospital. On hospital admission, the child’s temperature was 99.1°F (37.3°C) and a maculopapular rash on the face, neck, and trunk was noted, as well as buccal mucosal lesions. Subsequently, these buccal lesions were identified as Koplik spots by a consulting infectious disease specialist. However, there was a delay of approximately 5 hours before the child was moved to an airborne isolation room.

The following day (July 15), TCHD was notified of the suspected measles case and recommended that the child remain in airborne isolation during the remainder of the potential infectious period, 4 days after rash onset (i.e., through July 16). TCHD promptly activated their Public Health Incident Management Team to coordinate an urgent case investigation that involved contact identification, exposure assessment, and administration of PEP when appropriate, to prevent additional measles cases. The investigation required recruitment of public health investigators from TCHD and other state and local public health agencies in the Denver metropolitan area and assistance from hospital infection prevention specialists. Measles diagnosis was confirmed in the patient on July 16 by detection of measles virus by real-time polymerase chain reaction (PCR) from a nasopharyngeal swab specimen collected early on July 15; a positive measles immunoglobulin M (IgM) antibody titer was reported on July 20.

The patient’s period of infectivity, defined as 4 days before until 4 days after rash onset, extended from July 8 to 16. Potential exposures occurred at eight settings. Five settings (three health care facilities, an apartment building, and a children’s math and reading center) were deemed higher risk, based on exposure duration and proximity, and three (a supermarket, a large retail store, and a fast-food restaurant) were considered lower risk. A total of 311 possible contacts were evaluated from the higher risk settings. Among the 311 interviewed contacts, 283 (91%) were determined to have been potentially exposed and were evaluated for measles immunity (Table 1). According to the Advisory Committee for Immunization Practices (ACIP) recommendations for ascertaining presumptive immunity, persons who were born before 1957, had laboratory confirmation of immunity or prior measles disease, or had documentation of age-appropriate MMR vaccination were classified as immune (*1*). In addition, for this investigation, self-report of prior measles disease or MMR vaccination was used to classify persons as immune. Persons who were unable or unwilling to provide laboratory confirmation of immunity or were unsure about their measles disease or MMR vaccination history were classified as having unknown immunity, and persons who reported no previous receipt of MMR vaccine or measles disease were classified as susceptible. On the basis of these criteria, 244 (86%) of 283 potentially exposed persons were considered to be immune, seven (2%) had unknown immunity, and 32 (11%) were susceptible.

**TABLE 1:** Immunity status and public health response for contacts of two index measles cases — Colorado, 2016–2017

Immune status	Public health response	No. (%)	
		Patient A contact	Patient B contact
**Immune**	No action	244 (100)	161 (100)
	Subtotal	244	161
**Susceptible**	IG PEP with weekly follow-up*	22 (69)	0 (0)
	MMR vaccine PEP with weekly follow-up	9 (28)	0 (0)
	Quarantine^†^ with daily follow-up	1 (3)	1 (33)
	Exclusion from work for 21 days after exposure^§^	0 (0)	2 (67)
	Subtotal	32	3
**Unknown**	Weekly telephone follow-up	6 (86)	39 (57)
	Unable to contact; letters mailed if address known	1 (14)	20 (29)
	Out of state resident^¶^	0 (0)	9 (13)
	Subtotal	7	68
**Total contacts**		**283**	**232**

**Abbreviations:** IG = immune globulin; MMR = measles-mumps-rubella vaccine; PEP = postexposure prophylaxis.

* One person who received IG PEP could not be contacted for weekly follow-up.

^† ^Self-isolation at home.

^§^ One health care worker with receipt of one documented MMR vaccine dose and an equivocal measles Immunoglobulin G test, and one contact with a negative Immunoglobulin G titer were excluded from work.

^¶^ Information regarding these nine contacts was sent to relevant health departments that were responsible for follow-up.

During the 45-hour period after initiating the contact investigation, TCHD held two clinics to dispense PEP. PEP with MMR vaccine is recommended to prevent disease in exposed susceptible persons if exposure occurred within the preceding 72 hours, or with immune globulin (IG) if exposure occurred within 6 days and the susceptible person is at risk for severe illness from measles, which includes infants, pregnant women without measles immunity, or persons with severe immune system compromise. Among 32 susceptible contacts, 31 (97%) received PEP, including nine (28%) who received MMR vaccine and 22 (69%) who received IG (including two immunocompromised children and 15 infants aged <6 months). One susceptible contact was identified too late to receive PEP and was voluntarily quarantined at home and monitored daily for symptoms until the end of the incubation period. Susceptible contacts who received PEP and contacts with unknown immunity were monitored weekly for 21 days, the maximum incubation period.

To alert the public about the potential that lower risk exposures might have occurred in community settings, TCHD issued a press release on July 18 that advised anyone who had been in the facilities visited by the index patient during the period of infectivity to request MMR vaccination if they were not already immune to measles and to watch for symptoms.

No secondary cases of measles were identified among contacts, nor were any other cases of measles reported in Colorado within 4 months of the index case. However, an infant contact aged 8 months who had received MMR vaccine PEP experienced fever of 102.2°F (39°C) and diarrhea on July 20, 4 days after vaccination and 7 days after being exposed to the index patient. A maculopapular rash was reported on the torso on July 23 (7 days after MMR vaccination) and the infant experienced anorexia and irritability on July 24. Because TCHD was already monitoring the infant for symptoms, the infant was placed in home quarantine, and a nasopharyngeal swab was collected for measles real-time PCR testing, which was reported positive on July 28. The nasopharyngeal swab specimen was sent to the Viral and Rickettsial Disease Laboratory at the California Department of Public Health, where the measles virus was identified as genotype A, the MMR vaccine strain, indicating the infant’s febrile rash illness and positive measles real-time PCR was an adverse reaction to measles vaccine rather than a case of secondary transmission. In addition, the California Department of Public Health subsequently identified the measles genotype from the index patient as genotype B3, which is endemic in much of Africa and has been reported in India since 2012 (*2*).

## Patient B

On January 7, 2017 (approximately 6 months after the case in patient A), a second, unrelated measles case in an unvaccinated male adult aged 33 years was reported to public health in Denver, Colorado. The man had traveled to Thailand during November 20–December 14, 2016. The patient experienced a fever to 102.9°F (39.4°C) on December 20, followed by a coalescing macular rash on December 25, which started on the face, spread downward, and lasted for 8 days. The man was hospitalized during December 29–January 1; a blood sample collected on January 1 was reported as positive for measles IgM on January 6; TCHD was notified on January 7. During the infectious period (December 21–29), the patient visited 17 businesses and two health care facilities. The investigation protocol for patient A was used to classify contacts for patient B; however, in this investigation, contacts with only self-reported MMR vaccination were classified as having unknown immunity. Interviews with 248 possible contacts identified 232 (94%) who were potentially exposed and for whom measles immunity was assessed (Table 1). Among the 232 potentially exposed persons, three (1%) were susceptible to measles and either quarantined or excluded from work. Because public health was not notified of the case until >6 days from the time of exposure, PEP was not recommended. No secondary cases were identified.

TCHD prospectively tracked costs associated with these case investigations (Table 2). Personnel hours spent on the investigation were tracked in the agency’s human resources system, and costs were calculated based on individual salaries. TCHD’s nursing division provided costs for PEP supplies. Costs from external partners were requested and provided by each agency and stratified by personnel hours and supplies.

**TABLE 2:** Financial and personnel costs associated with investigation of two measles cases — Colorado, 2016–2017

Public health costs	Patient A investigation	Patient B investigation	Both investigations
Agencies involved (no.)	5*	3^†^	5*
Personnel time (hrs)	756	435	1,191
**Costs ($)**			
Personnel time and support^§^	35,339	17,868	53,207
MMR vaccine PEP	336	0	336
IG PEP	12,464	0	12,464
Laboratory costs	1,630	555	2,185
**Total costs**	**49,769**	**18,423**	**68,192**

**Abbreviations:** IG = immune globulin; MMR = measles-mumps-rubella vaccine; PEP = postexposure prophylaxis.

* The Tri-County Health Department (TCHD), Denver Public Health (DPH), Colorado Department of Public Health and Environment (CDPHE), and two health care facilities.

^†^ The TCHD, DPH, and CDPHE.

^§^ Personnel costs were calculated based on individual salaries multiplied by the number of hours spent on case investigation. TCHD included indirect costs. Only hours spent on public health investigation were included; other costs incurred at the hospital, including those related to direct patient care were not included. Personnel support costs included mileage and per diem. Personnel time estimates were tracked retrospectively for CDPHE.

For the first measles case investigation, efforts spanned three public health agencies and two health care facilities with 756 hours of personnel time dedicated to the incident, at a cost of $49,769. For the second case investigation, three public health agencies managed the investigation, which required 435 personnel hours at a cost of $18,423.

## Discussion

Measles is a highly infectious, vaccine-preventable viral disease that typically causes fever, cough, runny nose, conjunctivitis, and rash and can result in complications (otitis media, pneumonia, and encephalitis).

Endemic transmission of measles virus has not occurred in the United States since 2000 (*3*). U.S. outbreaks now typically occur when a traveler to a country with endemic measles transmission develops measles and the virus spreads in an undervaccinated community, amplifying the outbreak (*4*). A single case of measles prompts rapid case investigation, contact tracing, and use of PEP to prevent secondary transmission. Coordination from local and state public health agencies and health care facilities can improve timeliness of response and limit measles outbreaks.

This report highlights the high cost of public health response to measles introductions in local communities. Other published cost estimates of public health agency response to a single measles case range from $5,655 through $181,679 (*5*–*7*). Primary cost expenditures are personnel hours for contact tracing and coordination of PEP. The delay in reporting of patient B to public health precluded the use of PEP for contacts and resulted in lower costs. However, these missed opportunities for use of measles PEP could have led to secondary cases. 

The cost estimates of these two case investigations are pure cost estimates, without consideration of cost effectiveness. This is a limitation because it results in an underestimate of the true economic burden of these public health investigations.

In addition to the direct costs from personnel hours, these investigations place considerable burden on public health agencies. For example, the investigation for Patient A required support from 41 TCHD staff members representing disease control, environmental health, nursing, communications, emergency preparedness, and administration. Reprioritization of public health programming during these urgent investigations has the potential to cause delay in delivering other necessary public health services.

A febrile rash with typical onset 7–12 days after MMR vaccination occurs in approximately one in 20 vaccine recipients (*8*) and can be confused with secondary measles transmission from an index patient. Viral genotyping is recommended to distinguish between wild-type measles virus infection and a vaccine reaction.

Failure of clinicians to recognize measles early in the course of illness in these two cases serves as a reminder that health care providers might not be familiar with clinical measles or aware of the risk for measles transmission during international travel. Health care providers need to recommend MMR vaccination before travel when appropriate and maintain a high index of suspicion for measles in patients with a febrile rash illness, particularly unvaccinated returning international travelers. Because of ongoing measles transmission in other countries, importation into the United States will remain a threat. High population immunity, achieved through high 2-dose MMR vaccination coverage; prompt reporting of suspected measles cases to local public health agencies; and rapid diagnostic testing and implementation of local control measures are necessary to maintain measles elimination in the United States. Increased awareness by both clinicians and patients of international travel vaccination recommendations for measles is needed to prevent travel-associated measles infections. Even a single measles case can impose high economic and programmatic burdens on public health agencies.

**Summary**

**What is already known about this topic?**

Measles is a highly contagious, vaccine-preventable viral infection that has been eliminated in the United States. However, U.S. outbreaks typically occur when an international traveler introduces the infection to an undervaccinated community. Effective interruption of the outbreak requires timely and comprehensive case investigation by public health agencies.

**What is added by this report?**

During July 2016–January 2017, two single, unrelated measles cases were diagnosed in the Denver metropolitan area, each exposing hundreds of persons, prompting a complex and coordinated response by multiple public health agencies, costing in excess of $68,000.

**What are the implications for public health practice?**

Increased awareness of the risk of travel-associated measles infection is needed. Prior to international travel, measles-mumps-rubella vaccination is recommended to prevent measles disease. Even a single case of measles can cause substantial economic and personnel burden to public health systems. This burden can be decreased by improving measles-mumps-rubella vaccination rates, increasing timely reporting of suspected or confirmed measles cases, and optimizing coordinated public health response.

## Footnotes

* The Advisory Committee for Immunization Practices recommends routine childhood vaccination using measles-mumps-rubella (MMR) vaccine, with the first dose at age 12–15 months, and the second dose at age 4–6 years or at least 28 days after the first dose. International travelers aged ≥6 months are recommended to receive the MMR vaccine before travel.
